# *Ganoderma lucidum* Ethanol Extracts Enhance Re-Epithelialization and Prevent Keratinocytes from Free-Radical Injury

**DOI:** 10.3390/ph13090224

**Published:** 2020-08-29

**Authors:** Mario Abate, Giacomo Pepe, Rosario Randino, Simona Pisanti, Manuela Giovanna Basilicata, Verdiana Covelli, Maurizio Bifulco, Walter Cabri, Anna Maria D’Ursi, Pietro Campiglia, Manuela Rodriquez

**Affiliations:** 1 Department of Medicine, Surgery and Dentistry “Scuola Medica Salernitana”, University of Salerno, Via Salvatore Allende, 84081 Baronissi Salerno, Italy; mabate@unisa.it (M.A.); rrandino@unisa.it (R.R.); spisanti@unisa.it (S.P.); 2 Department of Pharmacy, University of Salerno, Via Giovanni Paolo II, Fisciano, 84084 Salerno, Italy; gipepe@unisa.it (G.P.); mbasilicata@unisa.it (M.G.B.); vcovelli@unisa.it (V.C.); dursi@unisa.it (A.M.D.); pcampiglia@unisa.it (P.C.); 3 Department of Molecular Medicine and Medical Biotechnologies, University of Naples Federico II, Via Pansini, 80131 Naples, Italy; maurizio.bifulco@unina.it; 4 Chemistry Department “G. Ciamician”, University of Bologna, Via Selmi 2, 33, 40126 Bologna, Italy; walter.cabri@unibo.it

**Keywords:** *Ganoderma lucidum*, wound healing, triterpenic acids, oxidative stress, cosmeceuticals

## Abstract

*Ganoderma lucidum* or Reishi is recognized as the most potent adaptogen present in nature, and its anti-inflammatory, antioxidant, immunomodulatory and anticancer activities are well known. Moreover, lately, there has been an increasing interest from pharmaceutical companies in antiaging *G. lucidum*-extract-based formulations. Nevertheless, the pharmacological mechanisms of such adaptogenic and regenerative actions remain unclear. The present investigation aimed to explore its molecular and cellular effects in vitro in epidermal keratinocyte cultures by applying liquid chromatography coupled to ion trap time-of-flight mass spectrometry (LCMS-IT-TOF) for analysis of ethanol extracts using ganoderic acid-A as a reference compound. The *G. lucidum* extract showed a keratinocyte proliferation induction accompanied by an increase of cyclic kinase protein expressions, such as CDK2 and CDK6. Furthermore, a noteworthy migration rate increase and activation of tissue remodelling factors, such as matrix metalloproteinases 2 and 9 (MMP-2 and MMP-9), were observed. Finally, the extract showed an antioxidant effect, protecting from H_2_O_2_-induced cytotoxicity; preventing activation of AKT (protein kinase B), ERK (extracellular signal-regulated kinase), p53 and p21; and reducing the number of apoptotic cells. Our study paves the path for elucidating pharmacological properties of *G. lucidum* and its potential development as cosmeceutical skin products, providing the first evidence of its capability to accelerate the healing processes enhancing re-epithelialization and to protect cells from free-radical action.

## 1. Introduction

*Ganoderma lucidum* (Ling Zhi (in China) or Reishi (in Japan)), also known as “the fungus of immortality”, is recognized among most important traditional medicinal mushrooms and most powerful adaptogens present in nature, since it acts as a regulator of biological functions [1,2,3]. The pharmacological properties of *G. lucidum*, such as anti-inflammatory, antioxidant, antiaging, immunomodulatory and antitumour activities [4,5,6,7], are due to its peculiar chemical composition in bioactive compounds such as polysaccharides, terpenoids, nucleotides, steroids, fatty acids, proteins and glycopeptides [8,9]. Based on that, *G. lucidum* was also reported to act as an adjuvant in the treatment of several diseases, i.e., anorexia, hypertension, insomnia and chronic hepatitis [10,11,12].

Among the terpenoid class, the most represented are triterpenes (ganoderic acids, ganoderoli acids, ganoderenics and lucid acids), which exhibit well-recognized anti-inflammatory, antioxidant, antitumour, anti-hepatitis, hypoglycaemic, antimalarial and antimicrobial activities [13,14,15,16,17,18,19].

Recently, bioactive extracts of *G. lucidum* have been having great success in the nutraceutical and cosmeceutical fields; indeed, its triterpenic acids are often found in cosmetics formulations [20]. In this respect, the *G. lucidum* extracts can be used to control hyperpigmentation as photoprotective agents, to suppress inflammatory skin diseases, to mitigate lipid metabolic disorders and to balance gut microbiota composition [21,22,23]. Nevertheless, the mechanism of action and the biological targets behind their beneficial dermatological effects are still unclear. Thus, a deeper understanding of the biology of the molecular interactions might be acquired.

Therefore, in this work, we aimed first to assess the chemical composition of triterpenic acids in the fruiting body of the fungus, highlighting its potential as a valuable source of bioactive compounds, and then to provide any further pharmacological evidence of how the ethanol extract of *G. lucidum* is able to boost the wound healing process and to prevent premature skin aging, lessening free-radical action. These findings paved the path for developing highly effective *G. lucidum-*based cosmeceuticals.

## 2. Results

### 2.1. Extraction and Characterization of G. lucidum Pericarp

**Figure 1:**
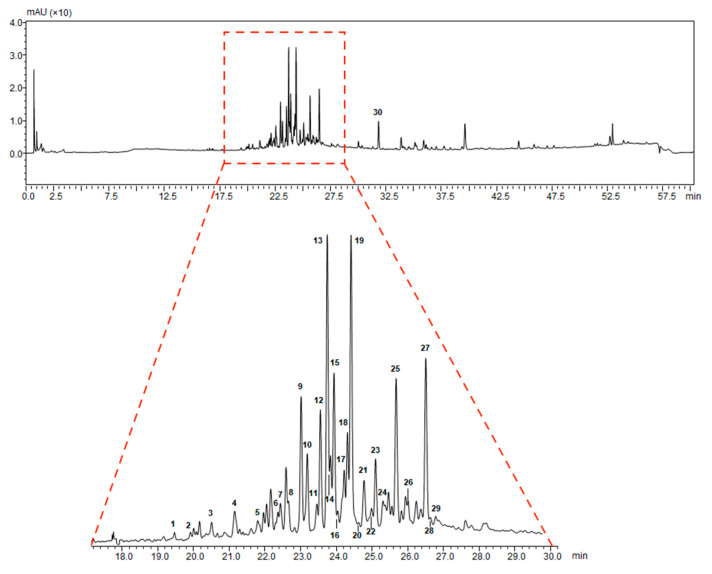
UHPLC-PDA (λ = 254 nm) chromatographic profile of the *G. lucidum* extract.

In the attempt to maximize ganoderic acid extraction, we performed an ethanol procedure operation as described in the Materials and Methods section. The ethanol extracts of *G. lucidum* were analysed by UHPLC-ESI-IT-TOF (Ultra-High Performance Liquid Chromatography-Electrospray ionization-Ion Trap-Time of flight) for quantification of triterpenes in *G. lucidum* ethanol extracts. In Figure 1, we showed the chromatographic profile acquired by UHPLC couplet to Photodiode Array Detector (PDA) (λ = 254 nm) of the *G. lucidum* extract.

In Table 1, we showed the chemical structures of the identified triterpenoids. In Table 2, identification and quantification of triterpenes in the *G. lucidum* ethanol extract were reported.

### 2.2. Evaluation of G. lucidum Extract Effect in HaCaT Cells

**Figure 2:**
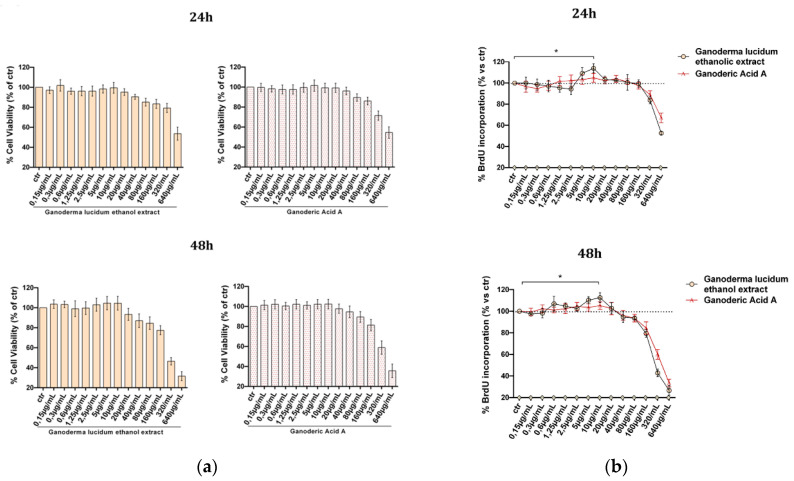
Evaluation of the *G. lucidum* extract effect in HaCaT cells: HaCaT cells were cultured for 24 or 48 h in the presence of the indicated concentrations (0–640 µg mL^−1^) of the *G. lucidum* extract or ganoderic acid A before MTT assay (**a**) or BrdU incorporation (**b**). The results are expressed as means ± SD of independent experiments performed in triplicate and str reported as percentage vs. the untreated control (ANOVA, * *p* < 0.05 vs. control).

First, we evaluated the effect of the extract on human keratinocytes. HaCaT cells were cultured with increasing concentrations of the ethanol extract and standard ganoderic acid A (0–640 µg mL^−1^) as a control for 24 and 48 h. As reported in Figure 2, only at the highest doses did the extract show a negative effect either on cell viability evaluated by MTT (3-(4,5-dimethylthiazol-2-yl)-2,5-diphenyltetrazolium bromide) assay (Figure 2a) or on cell proliferation (BrdU incorporation, Figure 2b), in both cases comparable to the pure ganoderic acid A profile.

Interestingly, we identified an interval of concentrations (5 and 10 µg mL^−1^) where the *G. lucidum* extract induced an increase of DNA synthesis, hence stimulating cell proliferation both at 24 h and 48 h (*p* ≤ 0.05).

### 2.3. Improvement of the Migratory Capacity of Human Keratinocytes Exposed to G. lucidum Extract

**Figure 3:**
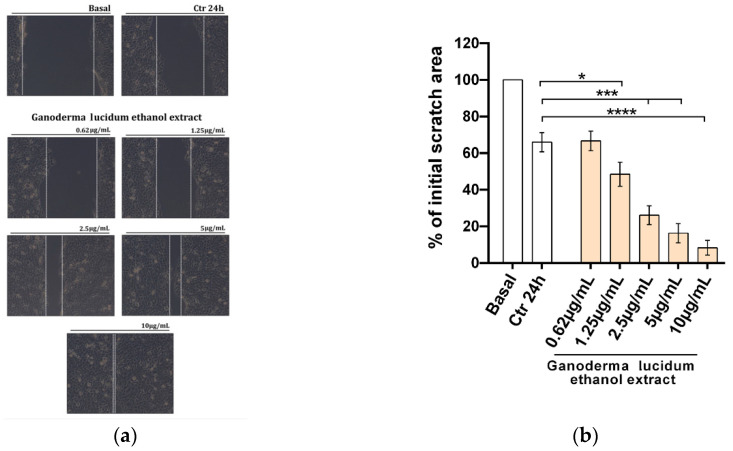
Improvement of the migratory capacity of human keratinocytes exposed to the *G. lucidum* extract: (**a**) wound healing assay performed in HaCaT cells treated for 24 h with vehicle (CTR) or *G. lucidum* extracts at the indicated concentrations (0.62–10 µg mL^−1^) in complete medium. Representative light microscope images from three independent experiments are shown. Dotted white lines indicate the wounded area from the initial scratch. Magnification is at ×20. (**b**) Histograms represent the mean scratch area observed in HaCaT cells expressed as a percent of the initial area. The measurement was made in three different experiments. The results are presented as mean ± standard error (ANOVA, * *p* < 0.05, *** *p* < 0.001 and **** *p* < 0.0001 vs. control).

In order to assess the potential effect of *G. lucidum* ethanol extract on the migratory function of HaCaT cells, we performed a scratch wound assay (Figure 3) treatment for 24 h with vehicle (CTR, control) or *G. lucidum* ethanol extracts at five increasing concentrations (0.62–10 µg mL^−1^) in complete medium (Figure 3a).

After 24 h of cell culture, in the presence of the *G. lucidum* extract, we observed an enhancement of wound healing at all doses tested from 0.62 to 10 µg mL^−1^, with 10 µg mL^−1^ being the most effective dose (****p < 0.0001), as shown in light microscope images from three independent experiments (Figure 3a) and in a histogram representation of the mean scratch area (Figure 3b).

### 2.4. G. lucidum Ethanol Extract Induces Expression of Proteins Linked to the Control of Cell Cycle and Migration

**Figure 4:**
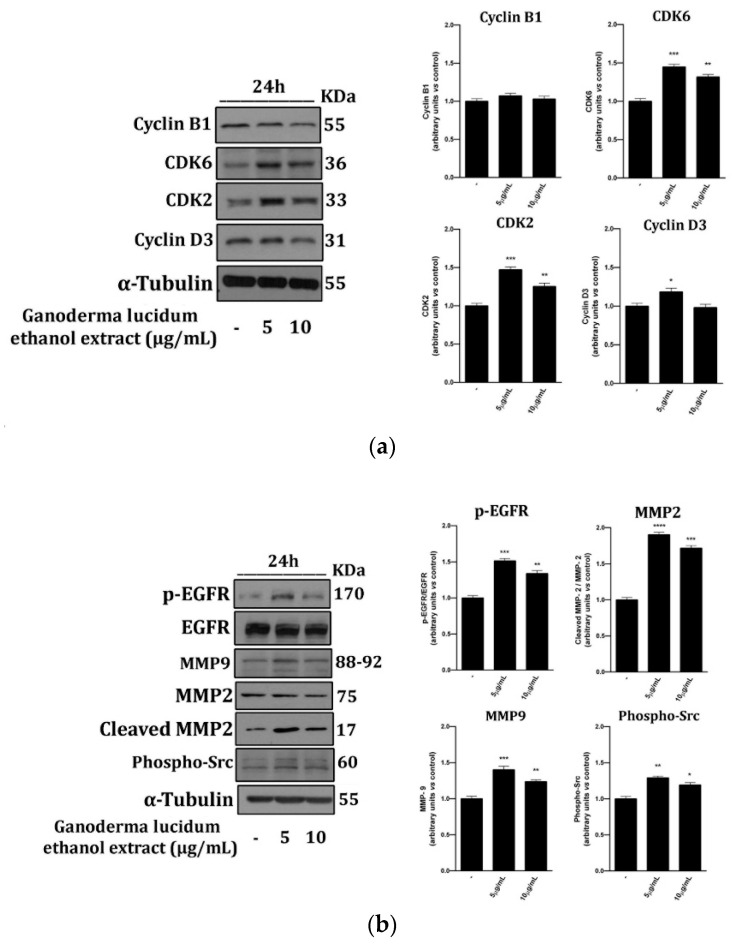
*G. lucidum* ethanol extracts induced cell cycle progression and migration protein expression. (**a**) Western blot analysis of cyclin B1, CDK6, CDK2 and cyclin D3 in whole cell extracts from HaCaT cells cultured for 24 h in the presence of the indicated concentrations of *G. lucidum* ethanol extract: Tubulin was used as a control for protein loading. The panel shows a representative Western blot of three different experiments performed with similar results. Histograms represent mean ± SD in densitometry units of scanned immunoblots from the 3 different experiments (ANOVA, *** *p* < 0.001, ** *p* < 0.01 and * *p* < 0.05). (**b**) Western blot analysis of p-EGFR (epidermal growth factor receptor)*,* EGFR, MMP-2 (total and cleaved), MMP-9 and Phospho-Src in whole cell extracts from HaCaT cells cultured for 24 h in the presence of the indicated concentrations of *G. lucidum* ethanol extract: Tubulin was used as a control for protein loading. The panel shows a representative Western blot of three different experiments performed with similar results. Histograms represent mean ± SD in densitometry units of scanned immunoblots from the 3 different experiments (ANOVA, **** *p* < 0.0001, *** *p* < 0.001, ** *p* < 0.01 and * *p* < 0.05).

In order to investigate the molecular pathways tuned by *G. lucidum* extracts in the previous experiments, we determined by Western blot analysis the status of the same proteins involved in both cell cycle progression and cell migration (Figure 4).

To this end, we treated cells with the *G. lucidum* ethanol extract at the most effective doses, 5 and 10 µg mL^−1^ for 24 h. According to the wound healing results, we observed that the *G. lucidum* ethanol extract increased the expression of cell cycle regulation proteins such as cyclin D3, CDK2 and CDK6 (Figure 4a). Besides, we showed that *G. lucidum* extracts induced MMPs, such as MMP2 (total and cleaved) and MMP9 expression (Figure 4b), and then subsequently triggered the EGRF signalling cascade. Activation of the downstream EGFR pathway, inducing phosphorylation of Src (Figure 4b), suggested that exposition to *G. lucidum* extracts provided a driving force in human keratinocyte migration.

### 2.5. G. lucidum Ethanol Extract Ameliorates Cytotoxicity and Apoptosis Induced by H_2_O_2_ in HaCaT Cells

**Figure 5:**
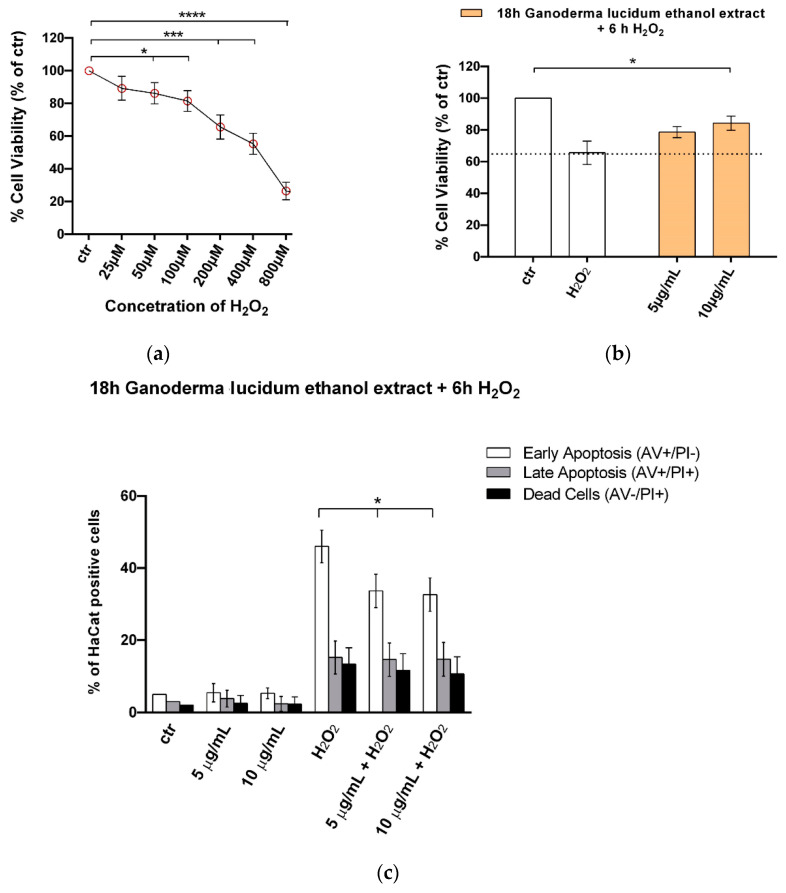
*G. lucidum* ethanol extract ameliorated cytotoxicity and apoptosis H_2_O_2_ induced in HaCaT cells: (**a**) HaCaT cells were cultured for 6 h in the presence of the indicated concentrations of H_2_O_2_ (25–800 µM) before MTT assay. The results are expressed as means ± SD of independent experiments performed in triplicate and are reported as percentage vs. the untreated control (ANOVA, * *p* < 0.05,*** *p* < 0.001 and **** *p* < 0.0001 vs. control). (**b**) HaCaT cells were cultured for 18 h in the presence of the indicated concentrations (0, 5 and 10 µg mL^−1^) of the *G. lucidum* ethanol extract before treatment with H_2_O_2_ for 6 h. The results are expressed as means ± SD of independent experiments performed in triplicate and are reported as percentage vs. the untreated control (ANOVA, * *p* < 0.05 vs. control). (**c**) Flow cytometric analysis of annexin V and propidium iodide (PI) double staining in the *G. lucidum* ethanol extract-treated HaCaT cells after 18 h and H_2_O_2_ for 6 h: histograms indicate the total percentage of early (annexin V-positive cells/PI-negative cells) and late apoptotic events (annexin V/PI-double positive cells) as well as necrotic cells (annexin V-negative cells/PI-positive cells). The results are representative of four independent experiments performed in duplicate and are expressed as mean ± SD (ANOVA, * *p* < 0.05).

HaCaT cells were exposed to H_2_O_2_ (0–800 µM) for 6 h, and the MTT assay was used as an indicator of cell viability (Figure 5). H_2_O_2_ induced cytotoxicity in a dose-dependent manner. The decrease in cell viability was statistically significant at 50 µM H_2_O_2_, whereas cell viability was reduced to 35.4% at 200 µM H_2_O_2_ (Figure 5a). Scientific evidence shows that relatively low concentrations of H_2_O_2_ caused apoptotic death of more cells (maximal at 250 µM), whereas 1000 µM H_2_O_2_ resulted in a reduction in apoptosis but an increase in overall cell death [24]. Therefore, in our system, we used 200 µM H_2_O_2_ in the subsequent experiments. We observed that at 18 h pretreatment of keratinocytes with the *G. lucidum* ethanol extract at 5 and 10 µg mL^−1^ protected the cells from H_2_O_2_-induced cytotoxicity. Cell viability declined to 35.4% ± 7.3% after exposure to 200 µM H_2_O_2_ for 6 h, whereas it increased to 78.6% ± 3.5% and 84.2% ± 4.5% with the *G. lucidum* extract at 5 and 10 µg mL^−1^ doses, respectively (Figure 5b). In order to strength the data obtained with the MTT assay, we performed a cell death analysis by annexin-V and propidium iodide double staining. As shown in Figure 5c, *G. lucidum* pretreatment before H_2_O_2_ exposure resulted in a significant reduction of apoptosis and particularly of early apoptosis.

### 2.6. G. lucidum Extract Prevents the Activation of Cell Death Molecular Pathways

**Figure 6:**
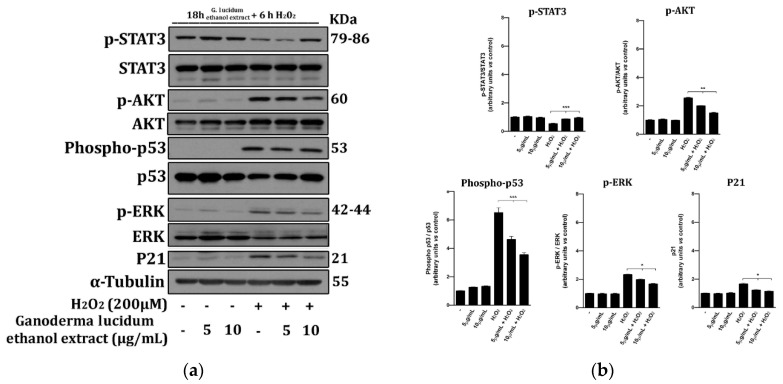
Ganonderic acid extract prevented the activation of cell death molecular pathways. (**a**) Western blot analysis of STAT3, AKT, p53, ERK (total and phosphorylated) and p21 in whole cell extracts from HaCaT cells cultured for 18 h in the presence of the indicated concentrations of the *G. lucidum* ethanol extract and H_2_O_2_ for 6 h: Tubulin was used as a control for protein loading. The panel shows a representative Western blot of three different experiments performed with similar results. (**b**) Histograms represent mean ± SD in densitometry units of scanned immunoblots from the 3 different experiments (ANOVA, *** *p* < 0.001, ** *p* < 0.01 and * *p* < 0.05).

AKT, ERK, p53 and p21 are critical proteins involved in the control of cell response to external damages, i.e., those induced by free radicals, and are involved in apoptosis activation [25,26]. Aiming to elucidate cell death molecular pathways modulated by *G. lucidum* extract, we performed Western blot analysis of STAT3 (Signal transducer and activator of transcription 3), AKT, p53, ERK (total and phosphorylated) and p21 in whole-cell extracts from HaCaT cells (Figure 6) using hydrogen peroxide to mimic oxidative stress-induced injury (OSI) within a short period [27].

The phosphorylation status of the abovementioned and p21 proteins in 18 h pretreated cells with ganoderic extract at 5 and 10 µg mL^−1^ doses, H_2_O_2_ or their combination was evaluated using tubulin as a control for protein loading (Figure 6).

We observed that H_2_O_2_ treatment increased the levels of p-AKT, p-ERK, phospho-p53 and p21 whereas *G. lucidum* extract treatment partially reversed these effects significantly, preventing both activation and shutdown of STAT3 signalling, critical for cell survival [28] (Figure 6a,b).

## 3. Discussion

Bioactive extracts from *G. lucidum* only recently have been reported to present remarkable in vitro and in vivo pharmacological properties beneficial also to the development of cosmeceutical formulations [21]. However, despite the commercial success, the pharmacological efficacy, especially in the field of cosmetic dermatology, still needs more in-depth scientific support. Lately, an in vitro analysis of the *G. lucidum* ethanolic extract as a dermatological ingredient was carried out, showing its suitability for skincare formulations as the absence of toxicity in keratinocytes and fibroblasts [28,29].

In this context, the present study reported the effect of *G. lucidum* on human keratinocytes as an in vitro skin model for evaluation of its dermatological properties which can be transferred to the cosmetics field for cosmetic use or to the therapeutic field for possible medical applications.

The ethanol extraction of *G. lucidum* was selected and listed as the most robust and suitable extraction method for this class of natural compounds [30,31], and detailed identification, characterization of chemical structures and quantification of triterpenes by UHPLC-ESI-IT-TOF analysis were performed for a detailed molecular description. In vitro studies on cultures of human keratinocytes were conducted by comparing *G. ludicum* extract activity with ganoderic acid A, the main *G. lucidum* described bioactive compound and mostly present in skin products available on the market [9,32,33]. Once the lack of cytotoxicity by *G. lucidum* ethanol extract was confirmed, we identified an interval of concentrations (5 and 10 µg mL^−1^) for the induction of cellular DNA synthesis, hence stimulating cell proliferation only for the *G. lucidum* ethanol extract, with respect to gandoreic acid A. This activity, observed only for the *G. lucidum* ethanol extract, might be ascribed to other bioactive ganoderic acids present in lower quantities or also might be attributable to a synergistic effect of various active components present in the ethanol extract.

Importantly, additional lines of evidence of this proliferation induction by *G. lucidum* ethanol extract treatment indicate an increase of the cyclin-dependent kinase protein expressions, mainly CDK2 and CDK6 (Figure 4a), key components of the cell cycle machinery for G1 to S transition.

To evaluate if *G. lucidum* ethanol extract treatment could be helpful for skin care, from minor, superficial and basic skin injuries to more complicated pathological states such as ulcers or bedsores, pathologies which, in addition to cell proliferation, also required suitable cell migration [34], we evaluated whether the extract treatment influenced the expression and activity of proteins involved in cell migration. Furthermore, we reported an increase of the migration rate supported by activation of the matrix metalloproteinases (MMPs), specifically MMP-2 and MMP-9; the pathways downstream EGFR stimulation; and phospho-Src (Figure 4b), important factors for stimulation of cell migration and normal tissue remodelling [35,36]. These results were confirmed by the functional wound healing assay at all doses tested from 0.62 to 10 µg mL^−1^, with 10 µg mL^−1^ being the most effective dose (Figure 3a,b).

Since oxidative stress plays a crucial role in several diseases pathogenesis, including allergic and inflammatory skin diseases like atopic dermatitis, urticaria and psoriasis [37], and in skin aging and since regulation of reactive oxygen species (ROS) levels is essential for maintenance of healthy skin homeostasis [38], we investigated whether the *G. lucidum* extract was able to protect cells from H_2_O_2_-induced cytotoxicity. In our system, 6 h exposure of 200 µM H_2_O_2_ reduced more than 35% of cell viability; thus, this concentration was used in subsequent experiments. The *G. lucidum* ethanol extract at 5 and 10 µg mL^−1^ doses reverted this trend, reducing the oxidative stress-induced injury to 15% (Figure 5b). These data were corroborated by annexin-V and propidium iodide double staining cell death analysis, which showed that *G. lucidum* pretreatment before H_2_O_2_ exposure resulted in a significant reduction of apoptosis, particularly of early apoptosis (Figure 5c). To elucidate cell death molecular pathways modulated by *G. lucidum* extract, we performed Western blot analysis of STAT3, AKT, p53, ERK (total and phosphorylated) and p21 in whole-cell extracts from HaCaT cells pretreated for 18 h with the ganoderic extract (Figure 6) using H_2_O_2_ to mimic OSI within a short time period. We observed that H_2_O_2_ treatment increased the levels of p-AKT, p-ERK, phospho-p53 and p21.

Our study confirmed that pretreatment with *G. lucidum* extracts partially reversed these effects, significantly preventing both activation and shutdown of STAT3 signalling involved in cell damage and apoptosis activation (Figure 6a,b). These data suggested that ERK signalling plays a critical role in the induction of survival, migration and proliferation in *G. lucidum* extract-treated cells and that protective effects against OSI, at least partially, depended upon STAT3 inhibition.

Our results, confirming what is already reported in scientific literature for the antioxidant activity of the *G. lucidum* extract [39] in addition to its anticancer, antimicrobial and anti-inflammatory activities [39,40], provide the first evidence of an increase in cell migration and an accelerated healing process and, at the same time, show the proteins involved and the possible molecular mechanisms in these activities.

## 4. Materials and Methods

### 4.1. Chemicals and Materials

The fruiting bodies of *G. lucidum* were provided by Indena S.p.A. (Viale Ortles 12, 20139 Milan, Italy) as dry material. Ganoderic acid A was purchased from Sigma-Aldrich Inc. (St. Luis, MO, USA). The *G. lucidum* extract was solubilized in dimethyl sulfoxide (DMSO) (0.01% in our assays) and added to cell cultures at the reported concentrations. H_2_O_2_ was purchased from Sigma-Aldrich (Milan, Italy). For Western blot analysis, the following antibodies were used: mouse monoclonal antihuman α-Tubulin, rabbit polyclonal antihuman phospho-STAT3 (p-STAT3; Tyr705), rabbit monoclonal antihuman STAT3, rabbit monoclonal antihuman phospho-p44/42 MAPK (p-Erk1/2; Thr202/Tyr204), rabbit monoclonal antihuman p44/42 MAPK (Erk1/2), rabbit monoclonal antihuman phospho-Akt (p-Akt; Ser473), rabbit monoclonal antihuman Akt, rabbit polyclonal p53, rabbit polyclonal antibody to phosphorylated p53, rabbit monoclonal antihuman p21, mouse monoclonal antihuman CDK6, rabbit monoclonal antihuman CDK2, mouse monoclonal antihuman cyclin D3, rabbit monoclonal antihuman phospho-EGFR (p-EGFR; Tyr1068) and rabbit monoclonal antihuman EGF receptor were purchased from Cell Signaling Technology (Danvers, MA, USA). Mouse monoclonal MMP2, mouse monoclonal antihuman MMP9, rabbit polyclonal antihuman Src (phospho Y418) and rabbit monoclonal antihuman cyclin B1 were purchased from Abcam (Cambridge, UK). Secondary HRP (Horseradish Peroxidase)-linked goat anti-mouse or goat anti-rabbit IgG were also purchased from Cell Signaling Technology (Danvers, MA, USA).

### 4.2. Sample Preparation

The dried powder of *G. lucidum* (3 g) was extracted with 100 mL of ethanol by refluxing in a Soxhlet apparatus for 6 h, and the solvent was evaporated under reduced pressure. The dried ethanol residue was then extracted with ethyl acetate three times. Finally, the ethyl acetate fractions were combined, filtered, evaporated and lyophilized for 24 h (LyoQuest-55, Telstar Technologies, Terrassa, Spain), using condenser temperature at −52 °C and 0.100 mBar as vacuum value [30].

### 4.3. LCMS-IT-TOF Analysis of G. lucidum Extract

A Shimadzu Nexera UHPLC system consisting of a SIL-30AC autosampler, a CBM-20A controller, a DGU-20 AR5 degasser, two LC-30AD pumps, a CTO-20AC column oven and an SPD-M20A photodiode array detector was used for UHPLC-ESI-IT-TOF analyses. The UHPLC system was coupled online to an LCMS–IT-TOF mass spectrometer through an ESI source (Shimadzu, Kyoto, Japan). LC-MS data elaboration was performed by the LCMSsolution^®^ software (Version 3.50.346, Shimadzu).

LC-MS analysis of the *G. lucidum* extract was carried out on Kinetex^®^ C18 150 × 2.1 mm (100 Å), packed with 2.6 μm core-shell particles column (Phenomenex, Bologna, Italy). The injection volume was 2 µL, and the flow rate was 0.5 mL min^−1^. The temperature of the column oven was set to 40 °C. The following PDA parameters were applied: sampling rate, 12.5 Hz; detector time constant, 0.240 s; and cell temperature, 40 °C. Data acquisition was set in the range 190–400 nm, and chromatograms were monitored at 254 nm at maximum absorbance of the compounds of interest. The mobile phase consisted of H_2_O (A) and ACN (B), both acidified by formic acid 0.1% *v*/*v*. The analysis was performed in gradient elution as follows: 0.01–5.00 min, isocratic to 1% B; 5.01–53.00 min, 1–95% B; 53.01–56.00 min, isocratic to 95% B; then four minutes for column re-equilibration.

Negative ionization mode was used for MS detection operating with the following parameters: detector voltage, 1.65 kV; CDL (Curved Desolvation Line) temperature, 250 °C; block heater temperature, 250 °C; nebulizing gas flow (N_2_), 1.5 L/min; and drying gas pressure, 100 kPa. Full scan MS data were acquired in the range of 150–1600 m/z (ion accumulation time, 30 ms; IT (Ion trap) repeat = 2). MS/MS experiments were conducted in the data-dependent acquisition, and precursor ions were acquired in the range 100–1200 m/z; ion accumulation time, 60 ms; CID (Collision Induced Dissociation) energy, 50%; collision gas, 50%; repeat = 1; execution trigger (BPC Base Peak Chromatogram) intensity, at the 70% stop level.

Identification was carried out based on standard retention time and UV spectra and by comparing MS/MS data with those present in the literature [30]. Molecular formulas were calculated by the Formula Predictor software (Version 1.12, Shimadzu), setting a low tolerance so that most of the identified compounds were in position 1 in the list of possible candidates.

### 4.4. Quantitative Analysis

As an external standard, we chose ganoderic acid A for quantification of triterpenes in the *G. lucidum* ethanol extract. The stock solution (1 mg mL^−1^) was prepared in ethanol, the calibration curve was obtained in a concentration range of 200–0.5 µg mL^−1^ with seven concentration levels (200, 50, 25, 10, 5, 1 and 0.5 µg mL^−1^) and triplicate injections of each level were run. Peak areas of ganoderic acid A were plotted against corresponding concentrations (µg mL^−1^). The amount of compounds in the sample was expressed as milligram per gram of dried extract, and linear regression was used to generate calibration curve with *r^2^* values ≥ 0.9999. Limits of detection (LOD) and quantification (LOQ) were calculated by the ratio between the standard deviation (SD) and the analytical curve slope multiplied by 3.3 and 10, respectively.

### 4.5. Cells

Human immortalized keratinocytes (HaCaT) were grown in Dulbecco’s modified Eagle’s medium (DMEM, GIBCO, Grand Island, NY, USA) and supplemented as described in detail elsewhere [41]. HaCaT cells were kindly provided by Giuseppe Monfrecola (Department of Experimental Dermatology, University of Naples, Naples, Italy).

All cell cultures were maintained at 37 °C in a humidified 5% CO_2_ atmosphere.

### 4.6. Determination of Cells Viability, MTT Assay

HaCaT cells (6 × 10^3^/well) were cultured for 24 h into 96-well plates before the addition of the individual substances at the indicated concentrations and were cultured for an additional 24–48 h at 37 °C. The reduction of the MTT (3-(4, 5-dimethylthiazolyl-2)-2, 5-diphenyltetrazolium bromide) tetrazolium salts assay was employed to examine cells’ viability, as described in detail elsewhere [42]. All experiments were performed in triplicate, and the relative cell viability was expressed as a percentage in comparison with the untreated control cells.

### 4.7. Determination of Cells Proliferation, BrdU Assay

HaCaT cells (6 × 10^3^/well) were cultured for 24 h into 96-well plates before the addition of the *G. lucidum* ethanol extract or ganoderic acid-A at the indicated concentrations and were cultured for an additional 24–48 h at 37 °C. Cell proliferation was evaluated by measuring BrdU incorporation into DNA (BrdU colorimetric assay kit; Roche Applied Science, South San Francisco, CA, USA) and was determined by an ELISA plate reader (ThermoScientific, Waltham, MA, USA) at 450 nm as described in detail elsewhere [43]. All experiments were performed in triplicate, and the relative cell growth was expressed as percentage in comparison with the untreated control cells (100%).

### 4.8. Scratch Wound Healing Assay

To evaluate the effect of *G. lucidum* extracts on HaCaT cell migration, the cells were plated in 6-well plates at a density of 5 × 10^3^ cells/well. When the confluent cells formed a homogeneous carpet and a vertical wound in the wells using a 200 µL tip was performed, culture medium containing *G. lucidum* extracts at the indicated concentrations or the vehicle alone was added to the wells, after the removal of detached cells. The wound area was recorded immediately and after 24 h through microscope analysis, as previously described [41].

### 4.9. Apoptosis Analysis

Quantitative assessment of apoptosis of HaCaT cells was analysed by antihuman annexin V (BioLegend, San Diego, CA, USA) using propidium iodide solution (PI) staining. Briefly, cells grown in 100-mm dishes for 24 h with *G. lucidum* extracts, H_2_O_2_ or combined as indicated were harvested with trypsin, washed in phosphate buffer saline (PBS) and subjected to apoptosis determination by the procedure described in detail elsewhere [43].

### 4.10. Western Blot Analysis

Cells were grown in p60 tissue culture plates at a density of 2 × 10^4^ cells/cm^2^ for 24 h. Cells were then incubated with vehicle, *G. lucidum* extracts (for 24 h), H_2_O_2_ (for 6 h) or their combination (*G. lucidum* extracts for 18 h and H_2_O_2_ for an additional 6 h), as indicated. After incubation, cells were washed with PBS, harvested and lysed in ice-cold RIPA lysis buffer (50 mM Tris-HCl, 150 mM NaCl, 0.5% Triton X-100, 0.5% deoxycholic acid, 10 mg mL^−1^ leupeptin, 2 mM phenylmethylsulfonyl fluoride and 10 mg mL^−1^ aprotinin) and then assayed for Western blot by the procedure, which is described in detail elsewhere [44].

### 4.11. Statistical Analysis

Statistical analysis was performed in all experiments shown by using the GraphPad Prism 6.0 software for Windows (GraphPad software). For each type of assay or phenotypic analysis, data obtained from multiple experiments are calculated as mean ± SD and analysed for statistical significance using the 2-tailed Student *t*-test for independent groups or using ANOVA followed by Bonferroni correction for multiple comparisons. *p* values less than 0.05 were considered significant. * *p* < 0.05, ** *p* < 0.01 and *** *p* < 0.001.

## 5. Conclusions

We provided the first scientific evidence that *G. lucidum* ethanol extract due to its high content in triterpenes remarkably increased cell migration patterns and accelerated the healing process, principally enhancing re-epithelialization and, at the same time, protecting the skin from the action of free radicals, paving the way for possible medical applications. Moreover, the prevention of skin aging leads to considering its formulations attractive as cosmeceuticals. Additional in vivo and clinical studies are requested to develop and validate novel nutraceuticals, cosmeceuticals and pharmacological formulations.

## Figures and Tables

**Table 1:** Chemical structures of the triterpenoids identified in the *G. lucidum* extract.

Peak	Retention Time (min)	Molecular Formula	[M − H]^−^ Observed	[M − H]^−^ Calculated	Error (ppm)	MS^2^ *m/z*	Tentative Identification
1	19.49	C_30_H_46_O_8_	533.3106	533.3120	−2.63	515.3096	12-hydroxyganoderic acid C_2_
2	19.94	C_30_H_42_O_8_	529.2807	529.2807	0.00	511.2468	20-hydroxyganoderic acid AM1
3	20.55	C_30_H_42_O_8_	529.2843	529.2807	4.89	511.2698	12-deacetylganoderic acid H
4	21.19	C_30_H_44_O_8_	531.2962	531.2963	−0.19	513.2930	Ganoderic acid η
5	21.75	C_30_H_42_O_8_	529.2774	529.2807	−6.23	511.2707; 467.2884	12-hydroxyganoderic acid D
6	22.42	C_30_H_46_O_7_	517.3189	517.3171	3.48	499.3131	Ganoderic acid C_2_
7	22.49	C_30_H_46_O_8_	529.2801	529.2807	−1.13	511.2640; 467.2661	Ganoderic acid C6
8	22.78	C_27_H_40_O_6_	459.2761	459.2752	1.96	441.2709	Lucidenic acid N
9	23.07	C_30_H_44_O_8_	531.2991	531.2963	5.27	513.2849; 469.2961	Ganoderic acid G
10	23.27	C_30_H_42_O_7_	513.2864	513.2858	1.17	495.2737	Ganoderenic acid B
11	23.52	C_30_H_44_O_7_	515.2979	515.3014	−6.79	497.2841	Ganoderic acid B
12	23.61	C_29_H_40_O_8_	515.2651	515.2650	0.19	473.2539	Lucidenic acid E
13	23.79	C_32_H_44_O_9_	571.2936	571.2913	4.03	553.2818	Ganoderenic acid K
14	23.85	C_30_H_42_O_7_	513.2847	513.2858	−2.14	495.2708	Ganoderic acid AM_1_
15	23.95	C_32_H_46_O_9_	573.3067	573.3069	−0.35	555.2977	Ganoderic acid K
16	24.09	C_30_H_42_O_8_	529.2825	529.2807	3.40	511.2753	Ganoderic acid derivative
17	24.25	C_32_H_42_O_9_	569.2750	569.2756	−1.05	551.2746	Ganoderic acid F
18	24.32	C_30_H_44_O_7_	515.3061	515.3014	4.12	497.2940	Ganoderic acid A
19	24.49	C_32_H_44_O_9_	571.2909	571.2913	−0.70	553.2850	Ganoderic acid H
20	24.68	C_30_H_40_O_8_	527.2648	527.2650	−0.38	509.2487	Elfvingic acid A
21	24.81	C_27_H_38_O_6_	457.2620	457.2596	5.25	439.2390	Lucidenic acid A
22	24.89	C_30_H_44_O_6_	499.3001	499.3065	−6.40	481.3798	Ganolucidic acid A
23	25.17	C_30_H_40_O_7_	511.2717	511.2701	3.13	493.2400	Ganoderenic acid D
24	25.31	C_27_H_36_O_6_	455.2449	455.2439	2.20	380.2072; 301.1813	Lucidenic acid F
25	25.72	C_29_H_38_O_8_	513.2508	513.2494	2.73	471.2400	Lucidenic acid D
26	26.04	C_34_H_46_O_10_	613.2997	613.3018	−3.42	595.2916; 553.2831	3-acetylganoderic acid H
27	26.58	C_32_H_42_O_9_	569.2736	569.2697	3.85	551.2649	12-acetoxyganoderic acid F
28	26.81	C_30_H_42_O_7_	513.2859	513.2858	0.19	451.2844	Ganoderic acid J
29	28.20	C_30_H_44_O_6_	499.3089	499.3065	4.81	437.2981	Ganolucidic acid D
30	31.98	C_30_H_44_O_5_	483.3122	483.3116	1.24	439.3309; 409.2717	Ganolucidic acid E

**Table 2:** Identification of triterpenes in the *G. lucidum* ethanol extract.

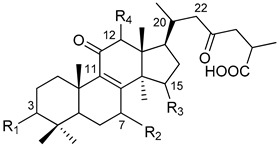		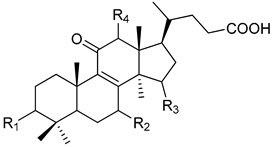			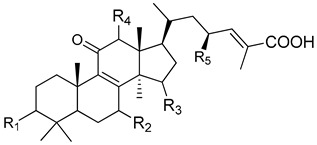			
A		B			C			
Peak	Compound Name	Type	R_1_	R_2_	R_3_	R_4_	R_5_	Double Bond
1	12-hydroxyganoderic acid C_2_	A	β-OH	β-OH	α-OH	OH	-	
4	Ganoderic acid η	C	β-OH	β-OH	=O	β-OH	β-OH	
5	12-hydroxyganoderic acid D	A	=O	β-OH	=O	OH	-	
6	Ganoderic acid C_2_	A	β-OH	β-OH	α-OH	H	-	
7	Ganoderic acid C6	A	β-OH	=O	=O	β-OH	-	
8	Lucidenic acid N	B	β-OH	β-OH	=O	H	-	
9	Ganoderic acid G	A	β-OH	β-OH	=O	β-OH	-	
10	Ganoderenic acid B	A	β-OH	β-OH	=O	H	-	Δ_20,22_
11	Ganoderic acid B	A	β-OH	β-OH	=O	H	-	
12	Lucidenic acid E	B	β-OH	=O	=O	β-OAc	-	
13	Ganoderenic acid K	A	β-OH	β-OH	=O	β-OAc	-	Δ_20,22_
14	Ganoderic acid AM_1_	A	β-OH	=O	=O	H	-	
15	Ganoderic acid K	A	β-OH	β-OH	=O	β-OAc	-	
17	Ganoderic acid F	A	=O	=O	=O	H	-	
18	Ganoderic acid A	A	=O	β-OH	α-OH	H	-	
19	Ganoderic acid H	A	β-OH	=O	=O	β-OAc	-	
20	Elfvingic acid A	A	=O	=O	β-OH	α-OH	-	Δ_20,22_
21	Lucidenic acid A	B	=O	β-OH	=O	H	-	
22	Ganolucidic acid A	A	=O	H	α-OH	H	-	
23	Ganoderenic acid D	A	=O	β-OH	=O	H	-	Δ_20,22_
24	Lucidenic acid F	B	=O	=O	=O	H	-	
25	Lucidenic acid D	B	=O	=O	=O	β-OAc	-	
26	3-acetylganoderic acid H	A	β-OAc	=O	=O	β-OAc	-	
27	12-acetoxyganoderic acid F	A	=O	=O	=O	β-OAc	-	
28	Ganoderic acid J	A	=O	=O	α-OH	H	-	
29	Ganolucidic acid D	C	=O	H	α-OH	H	β-OH	
30	Ganolucidic acid E	C	=O	H	α-OH	H	H	
